# Older adults in treatment for alcohol use disorders: service utilisation, patient characteristics and treatment outcomes

**DOI:** 10.1186/s13011-018-0176-z

**Published:** 2018-11-06

**Authors:** Hanna Dauber, Oliver Pogarell, Ludwig Kraus, Barbara Braun

**Affiliations:** 1 IFT Institut für Therapieforschung, Leopoldstr. 175, 80804 Munich, Germany; 2 Department of Psychiatry and Psychotherapy, Ludwig-Maximilians-Universität, Munich, Nußbaumstr. 7, 80336 Munich, Germany; 3 Department of Public Health Sciences, Stockholm University, 10691 Stockholm, Sweden; 4 Institute of Psychology, ELTE Eötvös-Loránd-University, Budapest, 1053 Hungary

**Keywords:** Older adults, Alcohol use disorder, Addiction care, Treatment, Early onset, Late onset

## Abstract

**Background:**

In western countries demographic changes are leading to an ageing society. Consequently, the number of older adults with alcohol use disorders (AUDs) will rise and the demand of treatment is likely to increase. However, thus far not many older adults with an AUD are seeking treatment and little is known about the efficacy of treatment for older adults. The present study aimed at determining the proportion of older adults with an AUD in addiction treatment, particular characteristics and treatment outcomes of this clientele.

**Methods:**

Using data of 10,860 patients with an AUD aged 60 and over that are documented within the national German addiction care system we conducted exploratory analyses with regard to prevalence, sociodemographic, disorder- and treatment-related variables.

**Results:**

Overall, we found a low proportion of older patients in treatment due to AUDs, but highly positive treatment outcomes. With regard to sociodemographic and disorder-related characteristics, older females and late-onset patients in particular constitute a unique clientele.

**Conclusions:**

The low service utilisation on the one hand but good treatment prognosis on the other emphasise the need to promote treatment seeking among older adults with AUDs. In this context, the special characteristics we found among older patients may contribute to better reach this population and to improve provisions of targeted treatment approaches.

## Introduction

In western societies a demographic change is taking place [1] that is leading to an ageing society. In 2015 the proportion of people in the German population aged over 60 was 27.4%, compared to 18.6% in 1964, and the proportion is expected to rise further in the coming years [2, 3]. With the growing number of older people in our society and the ageing ‘baby boomer’ generation, the number of older adults with alcohol use disorders (AUDs) is also expected to increase, thus posing a substantial public health challenge [4–7]. Accordingly, studies have reported a high prevalence of high risk alcohol consumption and AUDs among older adults [6, 8], while in general men are at higher risk than women [9–11]. In a German general population study, 17% of the 60–64 year-olds showed risky use of alcohol (> 12/24 g alcohol per day for women / > 24-60 g alcohol per day for men in the last 30 days), 18.5% reported episodic heavy drinking (more than five standard drinks of alcohol per day in the last 30 days) and 13.1% showed a clinical relevant use of alcohol (according to the Alcohol Use Disorder Identification Test, ≥ 8 points) [9]. About 2% fulfilled the DSM-IV (Diagnostic and Statistical Manual of Mental Disorders) criteria for an AUD (in the last 12 months) [12]. Although the widely varying definitions of problematic alcohol consumption make a comparison of study findings somewhat difficult, findings consistently show a relatively high prevalence of problematic alcohol use in Germany, compared to, for instance, the USA. There, 4.4% of the 60–64 year-olds showed heavy alcohol use (five or more drinks on the same occasion on each of 5 or more days in the past 30 days) and 9.7% report episodic heavy drinking [13]. While in general prevalence of episodic heavy drinking widely varies between countries, rates of AUDs are relatively similar [14, 15]. Overall, a conservatively estimated 400,000 people older than 60 years in Germany are affected by AUDs [5, 6, 12].

In marked contrast to the high and possibly increasing proportion of older adults with AUDs is the low utilisation of treatment within this group [6, 8]. Studies have demonstrated that, despite a good treatment prognosis, older adults rarely seek treatment for their AUD [5–7]. This may be due to shame, denial and lack of awareness of the problem or an inadequate health care provision for older adults [6, 16–18].

As the literature suggests, specific subgroups of older adults with AUDs exist with regard to age and circumstances at the onset of the AUD. Older people are often confronted with living conditions and experiences specific to late life which is characterised by loss of partners and friends, reduced or no involvement in the labour market, changes in financial circumstances, loss of social functions and, not least, lower mobility and poorer functioning [5, 17–19]. This in turn can lead to emotional and psychological problems such as loneliness, depression [17, 20], anxiety and sleep problems [17, 21], which can either foster or maintain an AUD. These age-specific risk factors may contribute to the late-onset of an AUD, while early-onset AUDs develop earlier in life and are closer related to general risk-factors such as low socioeconomic status, drinking behaviour and adverse social environmental factors [5, 17, 22, 23]. These differences suggest that when looking at older adults with AUDs, an examination of these subgroups should be included.

Although other countries have investigated large treatment populations [24], no comparable data are available in Germany, and little is known about addiction treatment and clinical characteristics of older adults with AUDs. Against this background the present study aims to determine service utilisation of older adults with AUDs as well as sociodemographic and clinical characteristics of this population and outcomes of addiction treatment for this group.

For this purpose, data from the German monitoring system of addiction care, which encompasses a multitude of heterogeneous treatment modalities, was investigated. These treatment modalities range from low-threshold services to case management, counselling services or rehabilitation in outpatient settings, to rehabilitation and detoxification in inpatient settings. Responsibility of drug treatment lies with the federal states and is funded by many different organizations, like the federal states, pension and health insurance bodies, municipalities, communities, charities, private institutions or companies. Most addiction care / counselling for drug users is provided in outpatient settings, which are often an entry point for clients and are provided free of charge. These facilities provide treatment either directly using their own resources or in collaboration with other institutions. Inpatient facilities for substance use disorders represent the second major pillar of drug treatment in Germany and are part of the medical system (for a comprehensive description of the organisation and provision of drug treatment in Germany see the Reitox report [25]). Patients treated within the addiction care system are documented within a national standardised monitoring system of addiction treatment centres in Germany that provides information on patient, disorder, and treatment characteristics in an aggregated form. Due to the large size of this nationwide treatment sample and good response rates an exploratory investigation provides representative and reliable results and gives valuable insight into patient, disorder-, and treatment-related variables.

Therefore, we analysed the data of patients aged 60+ years with an AUD in an exploratory design with regard to (a) the proportion of older adults with AUDs in addiction treatment, (b) sociodemographic and clinical characteristics of older adults with AUDs in addiction treatment, with a special focus on early- and late-onset patients and (c) treatment characteristics and treatment outcomes of older adults with AUDs. Overall, this study aims to provide a comprehensive overview of the current treatment situation for older adults with AUDs with regard to service utilization, characteristics and treatment outcomes.

## Methods

### Design and sample

Data from individuals within the German addiction care system (*n* = 837 outpatient centres, *n* = 206 inpatient centres), which are documented annually within the national standardised monitoring system (Statistical Report of Substance Abuse Treatment; DSHS; latest report: [26]), that is funded by the German Federal Ministry of Health to monitor and control addiction treatment, were analysed descriptively. Data are entered by treatment personnel and provide information on treatment facilities as well as sociodemographic, disorder- and treatment- related characteristics of patients. For data protection reasons, cases are aggregated on a facility level and are available in a bivariate cross-tabs format. No individual data are available. For further specifications of the methods see the DSHS report [26].

We analysed data of all patients aged 60+ years (60+) with a main substance diagnosis AUD (outpatient: *n* = 8598, inpatient: *n* = 2262) [27, 28], who have entered addiction treatment in 2014.

**Fig. 1:**
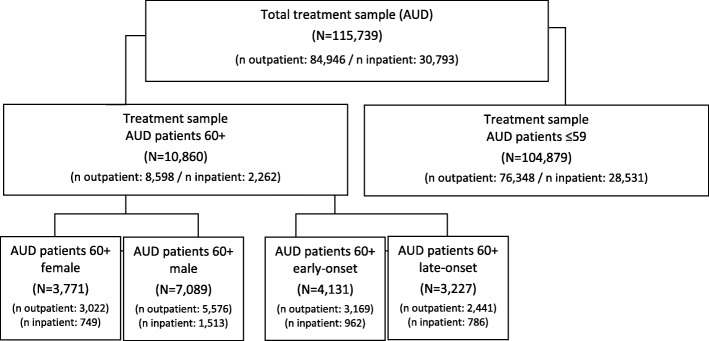
Overview of treatment samples. Total treatment sample of patients with AUD documented in the German Monitoring System of Substance Abuse Treatment is split into the treatment sample of older AUD patients aged 60+ years and the treatment sample of younger AUD patients (≤59). The treatment sample of older AUD patients aged 60+ years is further split into female and male patients and early- and late-onset patients. Beside the total number of each treatment sample numbers are also indicated for outpatient and inpatient settings. Because data of the age at onset of AUD are not available for all patients, subsamples of early- and late-onset patients do not add up to the number of the whole sample of AUD patients aged 60+ years

We used 60+ as a cut-off as this is a common threshold used to define “older adults” [5–7, 10–12, 16]. Additionally, as in Germany mean retirement age is about 60 years, it is indicated to use this age as a cut-off to cover all age-specific circumstances that are associated with this stage of life and which are of importance to answer our research questions. Within the German Monitoring System and in accordance to the German documentation system (see 2.2.) an AUD is documented as main diagnosis if in the last 12 months the ICD-10 (International Classification of Mental Diseases; [29]) diagnostic criteria for either harmful use of alcohol (F10.1) or alcohol dependence (F10.2) have been met and if this condition is the main reason for seeking help. Additionally, we analysed two datasets of AUD patients aged 60+ years with either early-onset (outpatient: *n* = 3169, inpatient: *n* = 962) or late-onset (outpatient: *n* = 2441; inpatient: *n* = 786) of the AUD (based on self-report) [30–33]. As in previous studies [22, 23] the cut-off age for late-onset was set at 45 years. For simplicity reasons patients with onset before 45 years old are referred to as early-onset patients, without further differentiation to match the incidence distribution of AUDs. For comparison, the sample of all outpatients (*n* = 76,348) and inpatients (*n* = 28,531) with an AUD below the age of 60 (**≤**59) (further referred to as younger sample / younger patients) was analysed. For detailed information on the sample composition see Fig. 1.

### Instruments

Data are documented within treatment centres, based on the German Core Data Set on the Documentation of Addiction Treatment (KDS; Deutscher Kerndatensatz zur Dokumentation in der Suchthilfe [34]), which is in accordance with the European TDI (Treatment Demand Indicator) [35] standard which measures drug treatment demand. Documentation is carried out via computerised software systems. The diagnostic criteria of the KDS are based on the ICD-10 [29] and documentation is intended to be carried out uniformly across treatment centres at the beginning and end of treatment.

All variables included in the analysis were operationalised according to the German documentation system: a) sociodemographic variables: age, gender, marital status (single, married – living together, married – living separately, divorced, widowed), living situation (alone, not alone), level of education (categories corresponding to German school levels) and employment status (regularly employed, retired), b) disorder-related variables: abstinence (in the last 30 days before entering treatment), age at onset of the AUD, duration of the AUD, comorbid substance use disorders (F11-F19 diagnosis according to ICD-10) and comorbid mental disorders (F0x, F2x-F9x diagnosis according to ICD-10), c) treatment-related variables: previous treatment, referral into treatment and d) ‘treatment outcomes’ which is used as a broader term for abstinence (in the last 30 days before end of treatment), treatment termination (regular/irregular, with “regular” comprising the regular termination of treatment, termination due to the instigation or with the consent of the therapist or the regular transition to another service, and “irregular” comprising premature termination due to disciplinary reasons, patient’s choice, unscheduled transition to another service or the death of the patient) and treatment outcome assessment at discharge (positive / negative, with “positive” comprising the assessment of the actual status of the AUD by the therapist at discharge as successful (remedy / abstinence) or improved (improvement of the AUD compared to the status at beginning of treatment) and with “negative” comprising the assessment of the status as steady (severity of the AUD remains unchanged) or impaired (severity of the AUD has deteriorated)). For a comprehensive description of all variables and item categories see the documentation manual [34].

### Analysis

Descriptive analysis was conducted using Microsoft Excel (2016). Due to structural differences between treatment settings within the German addiction care system (e.g., different resources, qualification of staff, modalities and type of treatment, legal frameworks and assignments), results are reported separately for outpatient and inpatient settings. Furthermore, sociodemographic, disorder- and treatment-related variables are reported separately for older males and females as well as for early- and late-onset patients. For comparison, results are also presented for the younger treatment sample of patients with an AUD younger than 60 years (**≤**59).

Due to the aggregated data format the use of common statistical tests for testing differences is limited. Furthermore, the use of chi^2^ tests is not recommended in such large sample sizes. Due to its high sensitivity, even small differences in the frequency distribution may reach statistical significance, without being clinically significant, misleading the interpretation of results [36]. Nevertheless, the widespread coverage of treatment centres in Germany [26] ensures highly representative results for all patients in German addiction care. Also, the fact that data collection is conducted within a standardised monitoring system of addiction care and not within a scientific study points to a high ecological validity of the data. Together with the large size of the data set, a descriptive analysis can be considered as sufficient and sensible. To enhance the validity of our findings, we only considered differences greater than 5 percentage points as clinically relevant.

## Results

### Number of older adults with an AUD in treatment

As demonstrated in Table 1, in outpatient settings 8598 AUD patients were over 60 years old. This accounts for 10.1% of all AUD outpatients. In inpatient settings 2262 AUD patients were over 60 years old, thus, 7.4% of all AUD inpatients.

**Table 1:** Sociodemographic characteristics by age, gender and early- or late-onset for patients in outpatient and inpatient treatment

	Outpatient treatment						Inpatient treatment					
	**≤**59^a^	60 +	60 + male	60 + female	60 + early^b^	60+ late^c^	**≤**59^a^	60 +	60 + male	60 + female	60 + early^b^	60+ late^c^
N	76,235	8598	5576	3022	3149	2425	28,338	2262	1513	749	962	786
Age (mean)	–	64.7	64.4	65.3	64.0	65.5	–	64.3	64.0	64.8	63.6	65.2
Sex female (%)	28.1	35.1	–	–	30.9	41.6	26.7	33.1	–	–	27.9	38.4
Marital status (%)												
Single (unmarried)	47.6	11.3	12.6	8.6	14.4	8.1	44.4	10.5	12.2	6.9	11.9	5.1
Married-living together	24.4	48.2	52.7	40.0	43.1	51.6	20.3	42.8	45.4	37.4	40.1	49.3
Married - living separately	6.2	5.8	6.4	4.8	6.2	5.6	8.6	9.3	10.5	7.0	9.7	8.6
Divorced	20.1	22.0	21.1	23.7	26.0	17.9	24.4	23.2	24.2	21.2	26.1	20.2
Widowed	1.7	12.8	7.3	22.9	10.3	16.8	2.3	14.2	7.7	27.5	12.2	16.9
Living situation alone (%)	43.6	41.6	37.0	50.0	44.8	40.1	56.4	49.2	46.8	54.4	49.5	43.8
Education high^d^ (%)	14.5	21.5	24.0	17.5	20.9	23.2	14.6	21.3	23.9	16.3	21.4	22.2
Employment												
Regularly employed (%)	49.6	23.5	25.0	20.5	24.6	22.0	43.0	27.4	29.5	22.8	26.8	24.5
Retired (%)	5.0	58.1	56.1	61.7	53.2	64.4	5.5	52.0	49.3	57.7	47.8	60.9

Definitions: ^a^**≤** 59 = sample of patients with an AUD in the German addiction care system under 60 years old; ^b^early = early-onset of the AUD (≤45 years); ^c^late = late-onset of the AUD (≥45 years); ^d^corresponding to A-levels

### Sociodemographic characteristics of older patients

The mean age of our older sample was 64.7 years in outpatient settings and 64.3 years in inpatient settings (see Table 1). On average, men were slightly younger than women and early-onset patients were younger than late-onset patients. While in the younger treatment sample women accounted for just one quarter of all patients, we found a higher proportion of women among older patients. The highest proportion of women was found among late-onset patients (outpatient: 41.6%, inpatient: 38.4%), which was markedly higher than among early-onset patients. Regarding marital status and living situation, about half of all older patients were married and living together with their partner, twice as many than in the younger sample. On the other hand, older patients were more often widowed, with the highest proportion among older women. Accordingly, older women lived alone more often than older men. Similar to older women, late-onset patients were also widowed more often than early-onset patients, whereas early-onset patients had a higher rate of divorce. About one quarter of the older patients, more than in the younger sample, showed a high educational level (corresponding to A-levels). The highest educational levels were found among older men. One quarter of the older patients were regularly employed (younger patients: outpatient: 49.6%, inpatient: 43.0%). This corresponds to the higher proportion of older patients in retirement. Older women and late-onset patients showed a retirement rate higher than older men and early-onset patients, respectively.

### Disorder-related characteristics

Regarding the frequency of alcohol use, 39.3% of older AUD outpatients and 64.8% of older AUD inpatients had been abstinent in the 30 days prior to entering treatment (see Table 2). The mean age at onset of the AUD among older patients was 40 years (in both settings). For men, AUD onset was on average five years earlier than for women. Among late-onset patients the mean age at AUD onset was 54.3 years in outpatient and 55.2 years in inpatient settings. Early-onset patients had a mean age of 29.8 (outpatient) and 28.3 years (inpatient) at AUD onset. Among older patients duration of the AUD was on average 25 years (in both settings) and men had been affected four to five years longer than women. As to be expected by definition, early-onset patients showed the longest duration of the AUD (outpatient: 34 years, inpatient: 35 years), whereas late-onset patients showed the shortest duration of the AUD (outpatient: 11 years, inpatient: 10 years).

**Table 2:** Disorder-related characteristics by age, gender and early- or late-onset for patients in outpatient and inpatient treatment

	Outpatient treatment						Inpatient treatment					
	**≤**59^a^	60 +	60 + male	60 + female	60 + early^b^	60+ late^c^	**≤**59^a^	60 +	60 + male	60 + female	60 + early^b^	60+ late^c^
Abstinence at treatment entry (%)	–	39.3	39.2	39.3	40.0	35.6	–	64.8	63.4	67.5	59.1	59.7
Age at onset of the AUD (mean)	–	40.3	38.5	43.4	29.8	54.3	–	40.1	38.2	43.8	28.3	55.2
Duration of the AUD (mean)	–	24.6	26.0	22.1	34.2	11.4	–	24.5	25.9	21.4	35.4	10.1
Comorbid substance use disorders (%)												
Tobacco	28.2	16.3	17.3	15.0	22.5	17.2	69.4	46.4	50.5	38.3	54.7	44.1
Opioids	2.4	0.5	0.5	0.4	0.6	0.3	4.5	1.0	1.0	1.0	1.6	0.5
Cannabis	7.9	0.4	0.5	0.2	0.5	0.6	15.5	1.0	1.3	0.4	1.5	0.4
Cocaine	2.4	0.1	0.1	0.0	0.2	0.0	5.1	0.4	0.6	0.1	0.7	0.3
Stimulants	4.4	0.1	0.1	0.1	0.2	0.1	9.5	0.2	0.2	0.3	0.2	0.1
Hallucinogens	0.7	0.1	0.1	0.1	0.1	0.3	2.2	0.1	0.1	0.1	0.2	0.0
Sedatives	1.6	1.6	1.1	2.4	1.7	1.9	4.0	4.1	2.7	6.9	4.6	4.6
Others	0.3	0.1	0.1	0.0	0.2	0.0	1.6	0.1	0.1	0.0	0.2	0.0
Comorbid mental disorders (%)	5.3	8.8	8.7	17.4	14.7	17.3	50.4	42.1	35.3	56.0	47.1	49.3

Definitions: ^a^**≤** 59 = sample of patients with an AUD in the German addiction care system under 60 years old; ^b^early = early-onset of the AUD (≤45 years); ^c^late = late-onset of the AUD (≥45 years)

In contrast to the younger treatment sample, where comorbid use of cannabis, cocaine and stimulants is more common, comorbid substance use disorders were relatively scarce in older patients. Among older AUD patients, only two substances were found to be used concurrent to alcohol in a problematic manner to a substantial extent: tobacco and sedatives.

In outpatient settings, 8.8% of the older patients had a documented comorbid mental disorder. In particular older women had a high prevalence of comorbid mental disorders (17.4%). In inpatient settings, comorbidity rates were generally higher than in outpatient settings, with 42.1% comorbid mental disorders among older patients. Again, older women had the highest prevalence of comorbid mental disorders.

### Treatment-related characteristics

In outpatient settings, one third of all older patients were in their first treatment episode, whereas in inpatient settings almost all patients (91.5%) had previously been in contact with (some kind of) addiction care (see Table 3). This is comparable to the proportion of re-treatment in the younger sample. In outpatient settings, late-onset patients showed a higher percentage of first treatment than early-onset patients. Regarding the ways of referral into addiction care, as in the you nger sample, most of the older AUD patients were self-referred in outpatient settings, with a higher rate for early-onset patients. Referrals by family, doctors/therapists or general hospitals account for another substantial proportion of referrals into outpatient settings. In inpatient settings, the majority of the older patients were referred by counselling facilities, with a higher rate for early-onset patients. Referrals by general hospitals account for another substantial proportion, with higher rates for late-onset patients. In both settings, almost none was referred by nursing homes.

**Table 3:** Treatment-related characteristics and treatment outcomes by age, gender and early- or late-onset for patients in outpatient and inpatient treatment

	Outpatient treatment						Inpatient treatment					
	**≤**59^a^	60 +	60 + male	60 + female	60 + early^b^	60+ late^b^	**≤**59^a^	60 +	60 + male	60 + female	60 + early^b^	60+ late^c^
First treatment (%)	33.1	31.0	32.3	28.9	25.4	36.7	9.8	8.5	9.0	7.3	8.4	9.9
Referral from (%)												
Self	39.8	40.1	39.7	40.8	43.9	36.6	1.8	2.9	2.7	3.2	2.0	4.1
Family	7.1	11.9	10.9	13.9	10.4	13.5	1.3	1.8	1.8	1.7	0.5	2.0
Doctor/Therapist	4.7	7.3	7.2	7.4	5.9	9.3	1.2	3.2	3.3	3.2	2.1	3.9
General hospital	12.4	16.2	15.9	16.7	15.2	16.5	19.3	22.3	21.5	24.1	19.5	27.5
Counselling	3.2	3.5	3.1	3.9	3.5	3.9	59.9	59.4	60.4	57.1	67.0	55.0
Rehabilitation	7.0	6.8	6.5	7.2	7.3	7.7	5.9	1.9	1.8	2.2	1.4	1.3
Nursing home	0.0	0.1	0.1	0.1	0.2	0.0	0.0	0.0	0.1	0.0	0.1	0.0
Others	25.8	14.1	16.6	10.0	13.6	12.5	10.6	8.5	8.4	8.5	7.4	6.2
Abstinence at discharge (%)	–	68.4	69.0	67.5	68.8	67.2	–	94.5	94.1	95.2	93.4	94.3
Treatment Termination regular (%)	67.3	77.5	77.7	77.4	77.5	79.3	84.5	90.0	89.5	90.9	90.7	90.3
Treatment outcome assessment positive (%)	67.6	74.6	74.5	74.8	73.6	76.7	83.8	89.3	89.4	88.8	89.7	90.1

Definitions: ^a^**≤** 59 = sample of patients with an AUD in the German addiction care system under 60 years old; ^b^early = early-onset of the AUD (≤45 years); ^c^late = late-onset of the AUD (≥45 years)

### Treatment outcomes

In the last 30 days before the end of treatment, about two thirds of the older outpatients and almost all older inpatients were abstinent. Thus, abstinence rates at discharge show an increase of about 30 percentage points compared to those at the beginning of treatment (Table 2). Related to treatment termination, older AUD patients showed a higher proportion of regular treatment termination (outpatient: 77.5%, inpatient: 90.0%) than the younger group. About three quarters of the older patients in outpatient settings and 89.3% in inpatient settings were assessed as successful at discharge and thereby surpassed treatment outcomes of the younger sample.

## Discussion

This study provides information on service utilisation of older adults with an AUD as well as on particular characteristics and treatment outcomes of older adults in addiction care. Altogether, we found a low proportion of older adults in treatment for AUDs but highly positive treatment outcomes at treatment discharge. Older women and late-onset patients constitute a unique clientele, regarding sociodemographic, disorder- and treatment-related characteristics.

### Access to addiction care and service utilisation among older adults

Although evidence suggests high treatment efficacy for older adults with AUDs [5, 6, 24] and German AUD treatment guidelines [37] recommend addiction treatment for older patients, the willingness of older people to utilise addiction care is low [6–8]. Based on the estimated number of 400,000 older adults with AUDs in Germany [5, 6, 12] our findings show that currently only about 3% of them are reached by addiction services. This is a much lower rate compared to the general adult population, where about 13–30% of those with an AUD seek some kind of addiction-related help [13, 38–42]. Keeping in mind that older adults account for about one quarter of the population but only for 7–10% of all AUD patients in addiction care, this affirms former notions of a significant underrepresentation of this population in addiction care [5, 6, 16, 18]. Consequently, this underlines the need for actions to better reach this clientele.

The fact that nearly all adults with an AUD (93%) are in contact with the primary care system [42] and considering the frequency with which older adults seek help for their multiple health problems, emphasises the important role primary care may play in the detection of AUDs and the initiation of adequate treatment [24, 43, 44]. The very low referral rates from medical doctors (and therapists) into addiction care, which have been found in this study, underline the need to intensify co-operations between addiction treatment facilities and primary care. Accordingly, the implementation of short screenings or special education and trainings for health practitioners may promote the recognition of AUDs among older adults in primary care settings [44]. Likewise, the low proportion of referrals by families reflects the finding that relatives of older adults with an AUD often do not address the problem due to shame, denial, misperceptions or even a lack of awareness of the alcohol abuse [16]. This emphasises the importance of promoting social environmental support (family-doctors, family, etc.) in encouraging help-seeking behaviour among older adults. Furthermore, the lack of referrals by age-specific institutions, such as nursing-homes, where alcohol misuse or dependence is also common [6] emphasises the need to focus on special services for older adults, and to establish networks and co-operations with these institutions which may help to reach this clientele.

Apart from these structural improvements health care utilisation might, amongst others, also be increased by the diversification of treatment approaches or a shift in treatment goals from abstinence-oriented treatment to moderate drinking. This may lead to an increased acceptance of addiction treatment and interventions [40]. Thus, especially in the case of less severe disorders, a return to moderate alcohol consumption might be an appropriate treatment goal.

### Characteristics of older AUD patients in treatment

The overall higher proportion of women among older patients with AUDs stands in marked contrast to the higher proportion of men with AUDs in the general population [10–12]. This may be explained by the higher proportion of women in this age group [45]. Otherwise, the high number of women with late-onset of an AUD may account for this divergent gender distribution. In our sample older women and late-onset patients were more frequently widowed and living alone than their male counterparts. This is in line with former findings that, among women, AUDs often develop later in life due to changes in life circumstances, such as the loss of a partner, or children leaving the parental home [46]. The high proportion of older patients in retirement reflects another specific living condition, which can result in a lack of social control or more time spent engaging in alcohol drinking activities, and thus contribute to the development of problem alcohol use in older age [16, 22]. Here again, women and late-onset patients might be more at risk, as in our sample, they showed less involvement in the labour market. The high educational level among older AUD patients in our sample is consistent with former findings [6, 10] which show a positive relation between educational level and alcohol consumption among older persons. On the other hand, seeking treatment seems also to be positively associated with educational level [47, 48] and could therefore explain the high educational levels in this treatment sample.

### Disorder- and treatment-related characteristics

Among persons with AUDs, seeking help seems to be influenced by the severity of the disorder [38–41]. Accordingly, persons with less severe AUDs often remain untreated [41]. With regard to severity of the AUD our findings seem to indicate that early-onset AUD patients show a longer and more complicated disorder- and treatment history and are rather comparable to younger patients with regard to their disorder-related characteristics. In contrast, late-onset AUD patients seem to be affected less severely with regard to a later AUD onset, shorter duration of the AUD and less previous treatment. This is in accordance with former findings, which found that late-onset AUD patients are affected less severely, even with regard to physical and psychical comorbid conditions [16, 22]. Generally, older patients show a lower burden of comorbid substance use disorders. Among older adults, only tobacco and sedatives use disorders were found comorbid with AUD. The latter is in accordance with the reported high use of medications among older adults [49]. The link between psychological problems and problem use of alcohol [20, 21, 50] is also evidenced in our sample by the high additional burden of comorbid mental disorders, especially among older women. In general, disorder-related characteristics seem to differ in particular between early- and late-onset AUD patients and not so much between age groups. The found differences between the different groups of older AUD patients may be important for tailored treatment approaches focusing on the unique characteristics of this populations.

### Treatment outcomes of older patients

With regard to abstinence at discharge, treatment adherence and treatment success, our findings confirm former studies which have found that older adults benefit highly from treatment, even more than younger patients [5, 6, 24, 37]. The proportion of regularly terminated treatment episodes among older patients is higher than in the sample of younger AUD patients and the majority of older patients’ outcomes were improved or successful at treatment discharge. This is also reflected in the abstinence rates which, at discharge, have almost doubled those at treatment entry. Accordingly, follow-up studies in German addiction care have found abstinence rates for AUD patients after one year between 41 and 79% in inpatient and 31–67% in outpatient settings [51, 52]. Thus, evidence indicates that the good treatment results among older adults remain relatively stable over time [51–53]. Contrary to former findings [5, 23, 24], we did not observe significant differences regarding the treatment outcomes of early- and late-onset patients.

### Limitations

Although the monitoring system of addiction care, with its large sample size, widespread coverage of treatment centres and detailed standardised documentation, offers a valuable base for exploratory descriptive investigations, the aggregated data format constitutes a limitation with regard to possible statistical analyses. Due to the high sensitivity of chi^2^ tests in such large sample sizes, descriptive analysis can be considered more sensible in this context and with regard to our study aim. For a test, we conducted *chi*^2^ tests for some of the variables, but, as assumed, all results were highly significant (e.g., “gender”: *chi*^2^: 695303.9042, *p* < .00001). For more sophisticated analyses, individual data need to be collected and investigated.

A further limitation lies in the operationalisation of treatment outcome assessment, which is only based on the subjective assessment by the therapist at discharge. No information is available on common outcome measures, such as rates of relapse or toxicological screenings at discharge or at a follow-up assessment. Integrating those measures when investigating large treatment populations would be an interesting subject for future research.

With regard to the specific study aim, it has to be mentioned that some examined variables are related to age and are likely to be confounded by cohort effects. Nevertheless, the aim of the study was not to quantify differences between younger and older patients with AUDs, but to gain insight into the older clientele and its characteristics. Another more critical confounder, which may explain the low number of older adults in alcohol treatment, lies in the decline of AUDs with age, due to the lower life-expectancy of people with AUDs and the high rate of deaths before age 65 [6]. Consequently, severity of disorder may also be influenced by this factor, as those who live beyond age 65 often show less severe disorders or have limited their consumption due to poor health, reduced tolerance or sequelae [6, 11]. Against this background the differentiation between early- and late-onset patients was important. Although recall bias is likely to pose a problem when it comes to investigating the exact age of AUD onset, evidence points to differences between groups of patients with early and late onset of the AUD. Thus, we examined patients with onset before and after the age of 45 years which resulted in two large samples: early-onset patients with a mean onset of the AUD at age 29 and late-onset patients with an onset at age 54. This also suggests that these two samples likely differ with regard to the circumstances that led to their problematic drinking.

## Conclusions

Altogether this investigation gives a broad overview of the current treatment situation of older adults with AUDs in German addiction care. Findings emphasise the importance of enhancing treatment utilization among older adults with AUDs. Despite the benefits of addiction treatment for older adults with AUDs, treatment utilization is still low. Thus, awareness of AUDs among older adults needs to be strengthened in our society and access to treatment needs to be facilitated. To be better prepared to treat this population and to offer adequate interventions, knowledge on older patients’ unique characteristics is essential.

The facts that, firstly, gender distribution in the treatment sample of older patients with AUDs is opposite to what we see in the general population, where men are three times more likely to have an AUD, and, secondly, older women in this sample differ in their characteristics and likely in their reasons for drinking, highlight the need to focus especially on treatment (and also research) of older women with AUDs. As findings show, women are living alone more often, showing higher rates of retirement and having more often a late-onset of the AUD, probably due to significant losses, while each of these is implying a lack of social contacts. Accordingly, an integration of social aspects into treatment for older women may be useful. Additionally, considering the high frequency of contacts with primary care among older adults, especially medical doctors may play an important role in the detection of AUDs and the initiation of adequate treatment. In this regard, the implementation of screenings, short interventions or special trainings for health practitioners may be useful.

In conclusion it seems worthwhile to make efforts to develop tailored treatment and access approaches to better reach this clientele, especially against the background of the positive treatment outcomes of older adults. Together with expanded co-operations with primary care and age-specific institutions this may serve to enhance treatment utilisation of older adults with AUDs.

## Abbreviations

AUD: Alcohol Use Disorder
DSHS: Deutsche Suchthilfestatistik (Statistical Report of German Substance Abuse Treatment)
DSM: Diagnostic and Statistical Manual of Mental Disorders
ICD: International Classification of Mental Diseases
KDS: Deutscher Kerndatensatz zur Dokumentation in der Suchthilfe (German Core Data Set for Documentation in Substance Abuse Treatment)
TDI: Treatment Demand Indicator
